# Development and validation of a prognostic model for post-surgical overall survival in Asian colon cancer patients: a real-world population-based study

**DOI:** 10.3389/fonc.2025.1541561

**Published:** 2025-04-17

**Authors:** Cheng Liu, Huaide Qiu, Junqiang Wang, Min Yang, Zhixiang Wang

**Affiliations:** ^1^ Department of Rehabilitation Medicine, Yixing No.2 People’s Hospital (Yixing Prevention and Treatment Hospital for Occupational Diseases), Yixing, Jiangsu, China; ^2^ School of Rehabilitation Science, Nanjing Normal University of Special Education, Nanjing, China; ^3^ Department of General Surgery, Yixing No.2 People’s Hospital (Yixing Prevention and Treatment Hospital for Occupational Diseases), Yixing, Jiangsu, China; ^4^ Rehabilitation Medicine Center, The First Affiliated Hospital of Nanjing Medical University, Nanjing, China

**Keywords:** overall survival, colon cancer, real-world study, Asian population, nomogram

## Abstract

**Objective:**

This study aimed to identify the determinants of postoperative overall survival in Asian patients with colon cancer and to establish a prognostic nomogram model.

**Methods:**

The study included colon cancer cases diagnosed between 2010 and 2015, sourced from the SEER database as well as an external cohort from Yixing No.2 People’s Hospital. Records with incomplete data on predetermined variables were excluded. The SEER dataset of eligible Asian postoperative colon cancer cases was split into a training set and a validation set with a 7:3 ratio. Prognostic factors affecting overall survival were identified using univariate and multivariate Cox regression analyses on the training set. A prognostic nomogram was developed with the R software package, and its predictive accuracy was evaluated in training, validation and external cohorts using ROC curves and calibration plots. Concordance index (C-index) and area under curves (AUCs) were also calculated, while decision curve analysis (DCA) was performed to examine the clinical utility.

**Results:**

Based on the criteria, 8738 cases from the SEER database were deemed suitable for analysis, and were divided into a training set (6118 cases) and a validation set (2620 cases) with a 7:3 ratio. An external cohort consisting of 73 cases with colon cancer was collected for external validation. The Cox regression analysis revealed that factors such as age, gender, marital status, histological type, grade classification, AJCC_T stage, AJCC_N stage, AJCC_M stage, CEA levels, and chemotherapy significantly influenced OS (P<0.05). These factors were incorporated into the nomogram, which demonstrated a C-index of 0.775 (95% CI: 0.766-0.784) for predicting OS in the training set, a C-index of 0.774 (95% CI: 0.760-0.787) in the validation set, and a C-index of 0.763 (95% CI: 0.698-0.828) in the external cohort. The nomogram was validated with good accuracy and clinical utility across three datasets.

**Conclusion:**

This study identified several independent prognostic factors influencing the postoperative overall survival of Asian colon cancer patients, including age, gender, marital status, histological type, grade classification, AJCC_T, AJCC_N, and AJCC_M stages, CEA levels, and chemotherapy. The constructed prognostic model showed good discrimination and accuracy, offering clinicians an individualized tool for survival predictions.

## 1 Introduction

Colorectal cancer (CRC) is a significant health burden worldwide, accounting for approximately 10% of all cancer diagnoses and deaths annually. It is the second most common cancer in women and the third in men, with men experiencing a 25% higher incidence and mortality rate (1, 2). In high-income countries, CRC incidence has either stabilized or declined, largely due to effective screening programs and lifestyle modifications, such as reduced smoking and increased aspirin use (3). However, despite these advances, early-onset CRC is increasing globally, posing new challenges for healthcare systems (3). Developed countries report the highest incidence rates, and projections suggest that global CRC cases could reach 2.5 million by 2035 (4).

Currently, many studies have analyzed prognostic factors by treating colorectal cancer (CRC) as a single cohort (5, 6). However, colon and rectal cancers exhibit distinct differences in terms of incidence, mortality, and patterns of distant metastasis. Research indicates that the incidence of colon cancer surpasses that of rectal cancer (7). Additionally, clinical outcomes and preferences for distant metastasis in colon cancer patients differ from those in rectal cancer patients (8). Consequently, it is essential to consider colon cancer patients as a distinct subgroup for more focused investigation. Surgical intervention is the cornerstone of colon cancer treatment. Advances in surgical techniques, particularly the adoption of laparoscopic surgery, have largely replaced traditional open procedures. Laparoscopic surgery offers numerous benefits, including shorter recovery times, fewer complications, and reduced postoperative pain (9–12). This minimally invasive approach allows for precise operations in multiple abdominal areas, minimizing tissue damage and lowering the risk of infection. In Japan, where colon cancer incidence is high, curative surgery is performed on a large majority of patients with stage I-III cancer, achieving five-year survival rates ranging from 62% to 91% (13). Postoperative survival in colon cancer is influenced by various factors, including race and ethnicity (14–17). Research indicates that survival outcomes can differ significantly among racial groups, yet there is a notable lack of studies focusing on Asian populations. This gap in research highlights the need for a deeper understanding of how clinical factors impact postoperative survival in Asian patients with colon cancer.

This study aims to address this critical gap by exploring the postoperative prognostic factors that affect Asian patients with colon cancer. Understanding these factors is essential for developing tailored treatment strategies and improving survival outcomes in this demographic. By focusing on the Asian population, this research seeks to contribute valuable insights into the unique challenges and considerations in managing colon cancer in Asian groups.

## 2 Methods

### 2.1 Data collection

The training and validation sets utilized in this study was sourced from the SEER database (18), a comprehensive research resource developed by the National Cancer Institute (NCI) in the United States. The SEER database serves as a crucial repository for cancer-related data, encompassing information on incidence, survival rates, and treatment outcomes across various regions in the U.S. This extensive dataset is maintained with rigorous quality control protocols, including systematic data review, cleansing, and validation processes, ensuring the data’s precision and reliability for supporting cancer research, epidemiological studies, and analyses of treatment efficacy and prognostic outcomes. Data access and extraction were facilitated using the SEER*Stat 8.4.3 software. An external cohort with colon cancer cases admitted to Yixing No.2 People’s Hospital from 2012 to 2024 was also collected. The retrospective collection and analysis of cases with colon cancer was approved by the Ethics Committee of Yixing No.2 People’s Hospital.

### 2.2 Inclusion and exclusion criteria

This model development focuses on cases diagnosed with colon cancer from 2010 to 2015, selecting only those records with complete data on specific variables. Key variables include the year of diagnosis, patient demographics such as age, gender, and race, marital status, tumor characteristics like grading and TNM staging per the 7th edition of the AJCC, tumor size, histological type, CEA levels, treatment details (radiotherapy and chemotherapy), primary tumor site, and survival duration.

Inclusion criteria require that patients were diagnosed with colon cancer between 2010 and 2015 and are above the age of 19. Participants must be identified as Asian or Pacific Islander, ensuring a focus on these specific populations. Essential data must be available, including marital status, TNM staging, tumor size, survival duration, and details on surgical intervention.The primary tumor site must be within the colon, specifically in the ascending colon, cecum, descending colon, hepatic flexure, sigmoid colon, splenic flexure, transverse colon, or rectosigmoid junction. Exclusion criteria eliminate records of patients with unknown marital status, TNM staging, tumor size, or survival time, or those with a survival time of less than one month. Additionally, cases with primary tumors located in the rectum or appendix, or those where no surgery was performed, are excluded.

### 2.3 Data processing and clinical characteristics profile

Patients were grouped based on specific information in the SEER database: patient age was divided into <50 years, 50-69 years, 70-79 years, 80+ years; gender was divided into: male, female; diagnosis time: 2010, 2011, 2012, 2013, 2014, 2015; marital status: married, SDW (single, divorced, separated, and widowed), and other marital statuses; histological type: adenocarcinoma, other types; SEER staging: early (Localized only), intermediate (Regional), late (Distant site(s)/node(s) involved); tumor location: cecum, ascending colon, hepatic flexure, transverse colon, splenic flexure, descending colon, sigmoid colon, rectosigmoid junction; tumor grading was divided into: G1, G2, G3-G4 stages; T stage (AJCC 7th edition) was divided into: Tis/T1, T2, T3, T4 stages; N stage (AJCC 7th edition) was divided into: N0, N1, N2; M stage (AJCC 7th edition) was divided into: M0, M1; whether radiotherapy was received and whether chemotherapy was received; carcinoembryonic antigen (CEA) level; tumor size: ≤30 mm, 31-60 mm, >60 mm. Overall survival (OS) was used as the prognostic assessment indicator. All eligible cases in the SEER database were included in the complete analysis set and then randomly divided into training and validation sets at a 7:3 ratio using the caret package (19). For continuous variables, the median and interquartile range of the three datasets were calculated. Using the training set, univariate and multivariate Cox regression analyses were conducted to determine the independent prognostic factors affecting the overall survival of Asian colon cancer patients after surgery.

### 2.4 Construction of the nomogram prediction model

Using the training set, univariate and multivariate Cox regression analyses were conducted to identify prognostic factors affecting the overall survival of Asian colon cancer patients after surgery. Univariate Cox regression is a classic method for identifying prognostic factors using survival data with time and event, but due to confounding effects, the selected prognostic factors may have false positives. To correct for confounding effects, multivariate Cox regression can be used for adjustment to identify the prognostic factors. These factors were used to construct a nomogram model for predicting the 1-year, 3-year, and 5-year overall survival rates of Asian colon cancer patients after surgery. The nomogram for prognostic factors was formulated using the “rms” package in R software.

### 2.5 Evaluation of the nomogram prediction model

Both the training and validation sets, along with the external cohort, were used to evaluate the prediction model. By comparing the nomogram-predicted survival rates with the actual survival rates in the training set, the area under the receiver operating characteristic (ROC) curve (AUC) was calculated to evaluate the classification ability (20), and calibration plots were drawn to assess the prediction accuracy (21). AUC>0.7 indicates good accuracy, while higher AUC values indicate better discriminatory ability. A validation method of 100 Bootstrap resamplings was used to draw calibration curves to evaluate the consistency between predicted and actual outcomes. If the point estimates and error bars are distributed near the diagonal line where predicted survival equals actual survival, the nomogram is considered accurate. ROC curves comparing the nomogram model and clinical characteristics were plotted to show difference in the discriminatory ability. Clinical utility was validated using decision curve analysis (DCA) (22), where higher curves indicate greater positive net benefits. Concordance index (C-index) was also calculated in the training, validation, and external cohorts, and comparisons among the published nomogram models were performed. The study with higher C-index value indicates better classification.

### 2.6 Statistical analysis

This study used R software for statistical analysis of the collected clinical data. Quantitative data were presented using median and interquartile range, while count data were presented using count and percentage. Univariate and multivariate analyses were performed using Cox regression methods, with results reported as hazard ratios (HR) and 95% confidence intervals to determine factors related to overall survival (OS) in Asian colon cancer patients. The “rms” package in R software was used to plot independent prognostic factors into a nomogram prediction model, to draw ROC curves, and calibration curves to evaluate the model classification and accuracy. P<0.05 indicates a statistically significant difference.

## 3 Results

### 3.1 Data processing and clinical characteristics profile

**Figure 1:**
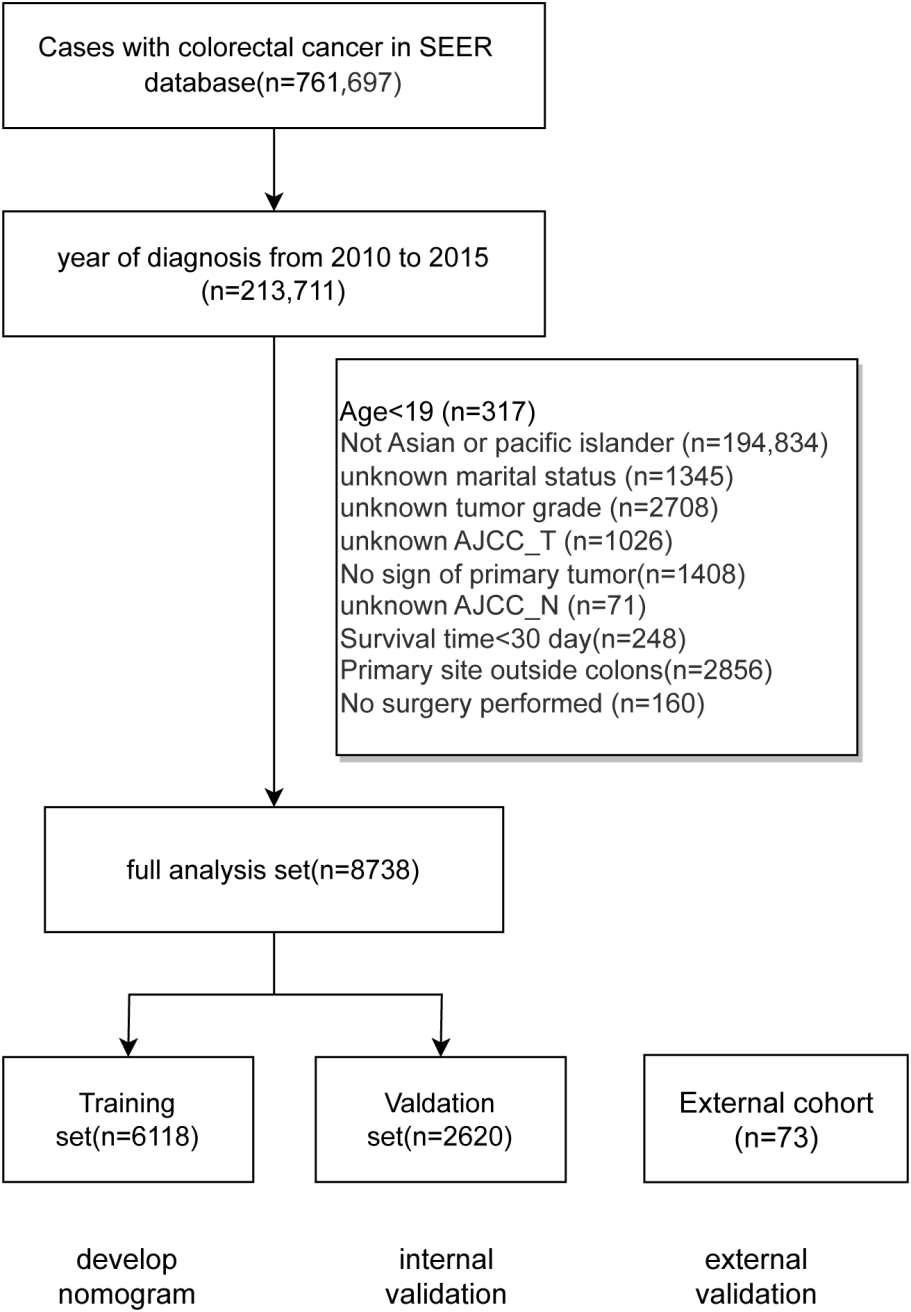
Data screening process and overall design.

The data screening process and study design were shown in **Figure 1**; a total of 8738 cases was included and subsequently split into a training set (6118 cases) and a validation set (2620 cases). Meanwhile, the basic characteristics of the external cohort with 73 cases were also presented. **Table 1** shows distribution of the demographic and clinicpathological variables. In the training cohort of this study, the age distribution revealed that most patients were diagnosed at an older age. Specifically, 26.19% were aged 60-69, and 23.08% were aged 70-79, while those aged 50-59 and over 80 constituted 19.65% and 18.88%, respectively. Patients under 50 represented a smaller segment at 12.21%. The gender distribution was nearly balanced, with females slightly outnumbering males at 50.83% compared to 49.17%. A significant majority, 64.94%, were married. The most common tumor locations were the sigmoid colon (33.10%), ascending colon (15.69%), and cecum (14.99%). Regarding tumor staging, a substantial 74.98% of patients were at Grade II, with T3 being the most prevalent in AJCC_T staging (54.00%) and M0 in AJCC_M staging (86.94%). All patients included after screening underwent surgical treatment, with adenocarcinomas comprising the vast majority (91.99%) of pathological findings. Tumor sizes varied: ≤30 mm accounted for 31.94%, 31-60 mm for 48.77%, and >60 mm for 19.29%. A minority of patients, 3.84%, received radiation therapy, whereas 37.40% underwent chemotherapy. The demographic and clinical characteristics of patients in the validation set mirrored those of the training group. The external cohort incorporated 73 cases with colon cancer admitted to Yixing No.2 People’s Hospital from 2012 to 2024. Detailed results for each prognostic variable are presented in **Table 1**.

**Table 1:** Clinical characteristics among different datasets.

Characteristic	Training	Validation	External
N	6118	2620	73
Survival_months	70 (35-96)	70 (35-96)	67 (35-98)
Age			
<50	747 (12.21%)	306 (11.68%)	4 (5.48%)
50-59	1202 (19.65%)	521 (19.89%)	21 (28.77%)
60-69	1602 (26.19%)	707 (26.98%)	17 (23.29%)
70-79	1412 (23.08%)	607 (23.17%)	17 (23.29%)
80+	1155 (18.88%)	479 (18.28%)	14 (19.18%)
Sex			
Female	3110 (50.83%)	1309 (49.96%)	33 (45.21%)
Male	3008 (49.17%)	1311 (50.04%)	40 (54.79%)
Year_of_diagnosis			
2010	991 (16.20%)	434 (16.56%)	
2011	999 (16.33%)	428 (16.34%)	
2012	1020 (16.67%)	410 (15.65%)	
2013	1009 (16.49%)	435 (16.60%)	
2014	1053 (17.21%)	467 (17.82%)	
2015	1046 (17.10%)	446 (17.02%)	
Marital_status			
Married	3973 (64.94%)	1716 (65.50%)	50 (68.49%)
SDW & other	2145 (35.06%)	904 (34.50%)	23 (31.51%)
Histologic_Type			
adenomas and adenocarcinomas	5628 (91.99%)	2430 (92.75%)	70 (95.89%)
other	490 (8.01%)	190 (7.25%)	3 (4.11%)
Site_recode			
Ascending Colon	960 (15.69%)	456 (17.40%)	13 (17.81%)
Cecum	917 (14.99%)	375 (14.31%)	11 (15.07%)
Descending Colon	490 (8.01%)	202 (7.71%)	1 (1.37%)
Hepatic Flexure	259 (4.23%)	96 (3.66%)	2 (2.74%)
Rectosigmoid Junction	753 (12.31%)	302 (11.53%)	11 (15.07%)
Sigmoid Colon	2025 (33.10%)	864 (32.98%)	28 (38.36%)
Splenic Flexure	219 (3.58%)	88 (3.36%)	1 (1.37%)
Transverse Colon	495 (8.09%)	237 (9.05%)	6 (8.22%)
SEER_Stage			
Localized only	2337 (38.20%)	966 (36.87%)	
Regional	2920 (47.73%)	1280 (48.85%)	
Distant site (s)/node (s) involved	861 (14.07%)	374 (14.27%)	
Grade			
I	461 (7.54%)	175 (6.68%)	6 (8.22%)
II	4587 (74.98%)	1938 (73.97%)	54 (73.97%)
III-IV	1070 (17.49%)	507 (19.35%)	13 (17.81%)
AJCC_T			
Tis/T1	817 (13.35%)	332 (12.67%)	13 (17.81%)
T2	858 (14.02%)	359 (13.70%)	45 (61.64%)
T3	3304 (54.00%)	1422 (54.27%)	6 (8.22%)
T4	1139 (18.62%)	507 (19.35%)	9 (12.33%)
AJCC_N			
N0	3215 (52.55%)	1349 (51.49%)	42 (57.53%)
N1	1718 (28.08%)	808 (30.84%)	17 (23.29%)
N2	1185 (19.37%)	463 (17.67%)	14 (19.18%)
AJCC_M			
M0	5319 (86.94%)	2269 (86.60%)	66 (90.41%)
M1	799 (13.06%)	351 (13.40%)	7 (9.59%)
Radiation			
No	5883 (96.16%)	2527 (96.45%)	71 (97.26%)
Yes	235 (3.84%)	93 (3.55%)	2 (2.74%)
Chemotherapy			
No	3830 (62.60%)	1635 (62.40%)	45 (61.64%)
Yes	2288 (37.40%)	985 (37.60%)	28 (38.36%)
CEA			
0	2061 (33.69%)	919 (35.08%)	26 (35.62%)
unknown	2247 (36.73%)	932 (35.57%)	20 (27.4%)
1	1810 (29.58%)	769 (29.35%)	27 (36.99%)
CS_tumor_size			
<=30	1954 (31.94%)	854 (32.60%)	21 (28.77%)
31-60	2984 (48.77%)	1277 (48.74%)	42 (57.53%)
>60	1180 (19.29%)	489 (18.66%)	10 (13.7%)
Vital_status			
0	3570 (58.35%)	1542 (58.85%)	35 (47.95%)
1	2548 (41.65%)	1078 (41.15%)	38 (52.05%)

### 3.2 Identification of prognostic factors

Using the training set, we applied both univariate and multivariate Cox regression models to assess the impact of various variables on postoperative overall survival (OS) in Asian colon cancer patients. As shown in **Table 2**, radiation therapy did not show significant differences between groups in terms of OS. However, other variables including age, sex, marital status, histologic type, chemotherapy, Grade classification, AJCC_T stage, AJCC_N stage, AJCC_M stage, CEA level, and tumor size (mm) demonstrated significant differences, indicating they are potential prognostic factors for postoperative outcomes in this patient population. The multivariate Cox regression analysis provided several significant insights into the prognostic factors influencing overall survival (OS) in Asian colon cancer patients. Age emerged as a crucial factor, with patients aged 70-79 and those over 80 showing significantly lower OS compared to individuals aged 50 and below. Gender differences were also notable, as male patients exhibited significantly different OS compared to their female counterparts. Marital status contributed to distinct survival chance on colon cancer patients post-surgery, with single, divorced, or widowed individuals experiencing significantly inferior OS compared to married patients.

**Table 2:** Identification of prognostic factors using cox regression.

	Univariate cox					Multivariate cox			
	Ref	HR	HR.95L	HR.95H	pvalue	HR	HR.95L	HR.95H	pvalue
Age	<50								
	50-59	0.84	0.72	0.99	0.042	1.07	0.90	1.26	0.444
	60-69	0.99	0.85	1.15	0.856	1.28	1.10	1.50	0.001
	70-79	1.48	1.28	1.72	<0.001	1.99	1.71	2.31	<0.001
	80+	2.86	2.48	3.30	<0.001	3.91	3.35	4.55	<0.001
Sex	female								
	male	1.18	1.09	1.27	<0.001	1.34	1.24	1.45	<0.001
Marital_status	Married								
	SDW or other	1.32	1.22	1.43	<0.001	1.14	1.05	1.24	0.002
Histologic_Type	adenocarcinoma								
	other	1.51	1.33	1.71	<0.001	1.19	1.04	1.35	0.011
Grade	I								
	II	1.65	1.38	1.98	<0.001	1.20	0.99	1.44	0.058
	III-IV	2.75	2.26	3.33	<0.001	1.50	1.22	1.83	<0.001
AJCC_T	T1								
	T2	1.30	1.07	1.58	0.009	1.01	0.83	1.24	0.888
	T3	2.33	1.99	2.73	0.000	1.33	1.11	1.60	0.002
	T4	4.96	4.20	5.85	<0.001	2.27	1.87	2.77	<0.001
AJCC_N	N0								
	N1	1.72	1.56	1.89	<0.001	1.53	1.38	1.70	<0.001
AJCC_N	N2	3.24	2.95	3.56	<0.001	2.41	2.15	2.71	<0.001
AJCC_M	M0								
	M1	5.01	4.58	5.49	<0.001	3.87	3.50	4.29	<0.001
Radiation	No								
	YES	1.02	0.84	1.24	0.821				
Chemotherapy	No								
	YES	1.15	1.06	1.25	<0.001	0.67	0.61	0.74	<0.001
CEA	0								
	unknown	1.37	1.24	1.52	<0.001	1.26	1.14	1.39	<0.001
	1	2.34	2.12	2.59	<0.001	1.58	1.43	1.76	<0.001
CS_tumor_size	≤30								
	31-60	1.74	1.58	1.91	<0.001	1.07	0.96	1.20	0.197
	>60	1.92	1.71	2.15	<0.001	1.00	0.88	1.14	0.977

Tumor grade proved to be significant; Grades III-IV were associated with different OS compared to Grade I. The stage of cancer, particularly AJCC_T stage T3 and T4, was significantly linked to OS, contrasting with Tis/T1 stages, while T2 stages did not show significant differences. Lymph node involvement was another critical factor, with AJCC_N stages N1 and N2 significantly associated with reduced OS compared to N0. Additionally, the presence of metastasis, as indicated by AJCC_M stage M1, was significantly associated with OS compared to M0. The analysis further revealed that patients who received chemotherapy had significantly increased OS compared to those who did not. CEA levels were also indicative of prognosis, with unknown and elevated levels significantly associated with OS compared to normal levels. Interestingly, tumor size did not show significant differences in OS, whether the size was 31-60 mm or greater than 60 mm, compared to tumors 30 mm or smaller. This could be due to the interaction between tumor size and AJCC_T stage, where the survival difference was already explained by AJCC_T stage. The study identifies age, gender, marital status, chemotherapy, tumor grade, AJCC_T stage, AJCC_N stage, AJCC_M stage, and CEA levels as independent prognostic factors affecting postoperative outcomes in Asian colon cancer patients. Detailed results are presented in **Table 2**.

### 3.3 Construction and validation of a nomogram model

**Figure 2:**
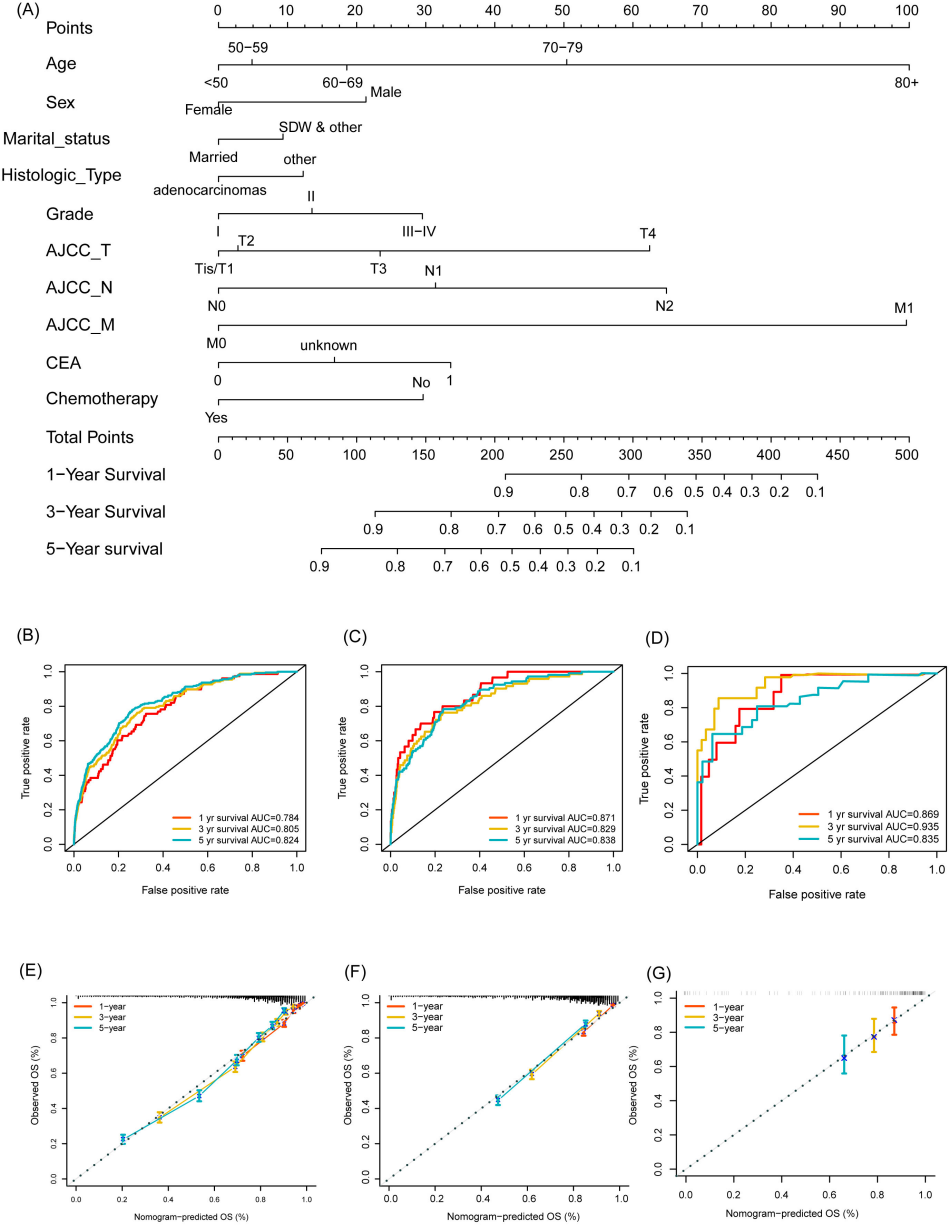
Development and validation of a nomogram for predicting overall survival in Asian individuals with colon cancer. **(A)** Nomogram for predicting overall survival probabilities; **(B–D)** ROC curves for 1-year, 3-year, and 5-year survival predictions in the training set **(B)**, validation set **(C)**, and external cohort **(D)**; **(E–G)** Calibration plots for 1-year, 3-year, and 5-year survival predictions in the training set **(E)**, validation set **(F)**, and external cohort **(G)**.

**Figure 3:**
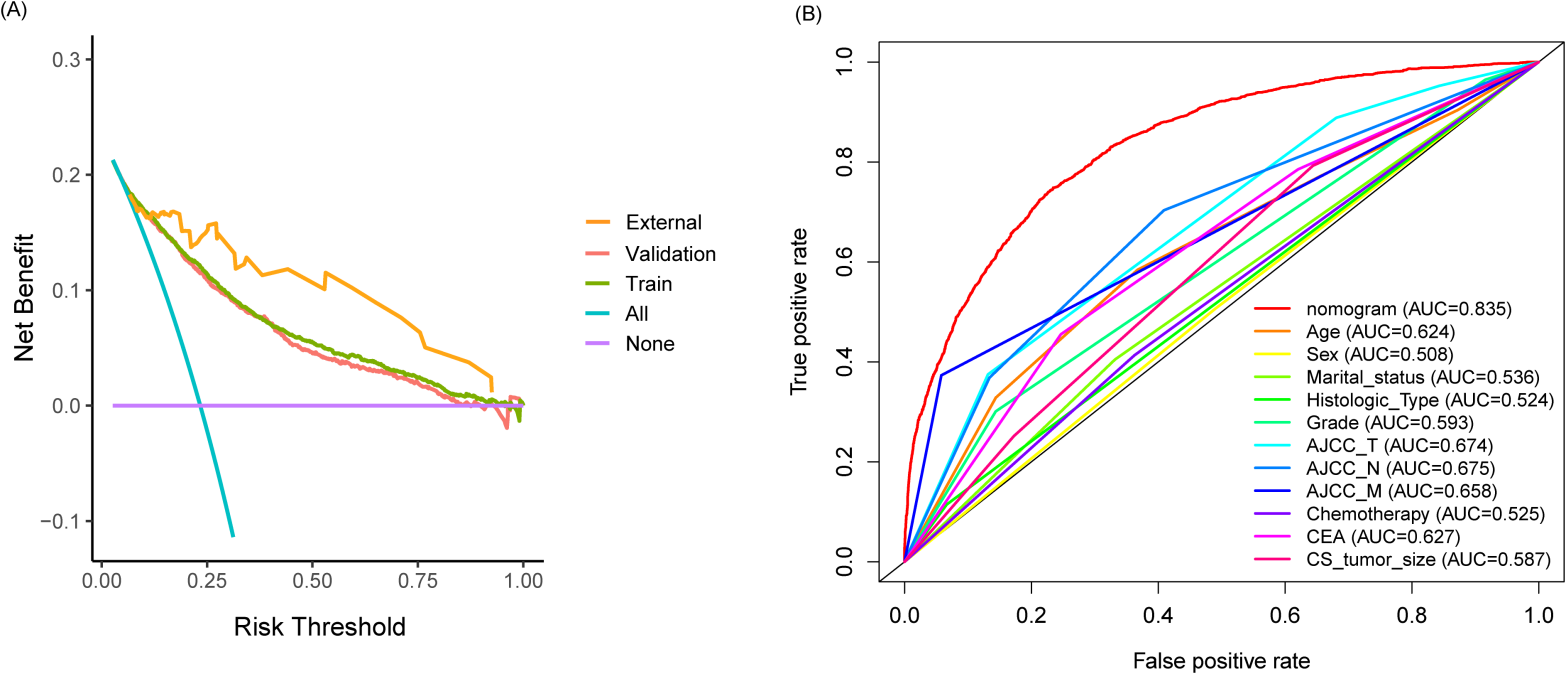
Evaluation of clinical utility in the nomogram for predicting outcomes. **(A)** Decision curve analysis (DCA) comparing the net benefits of the nomogram in the training set, validation set, and external cohort; **(B)** ROC curve comparing the discriminatory power of the nomogram and various individual clinical predictors.

**Figure 4:**
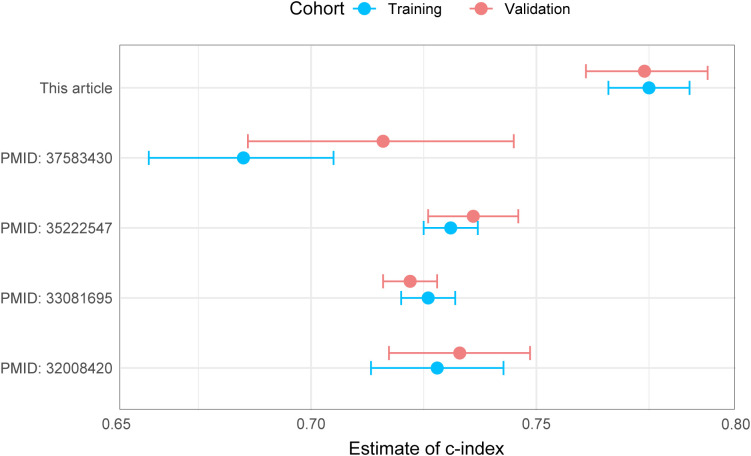
Comparison of C-Index among published nomogram models.

As shown in **Figure 2A**, the nomogram applies patient characteristics identified as prognostic factors to predict 1-year, 3-year, and 5-year survival probabilities. Each variable is assigned points, and the total points indicate the individual survival probabilities. The nomogram demonstrated a C-index of 0.775 (95% CI: 0.766-0.784) for predicting OS in the training set, a C-index of 0.774 (95% CI: 0.760-0.787) in the validation set, and a C-index of 0.763 (95% CI: 0.698-0.828) in the external cohort. Subsequently, the model’s classification performance was then validated using ROC curves in the training, validation, and external cohorts. The AUC values for 1-year, 3-year, and 5-year survival predictions are: 0.784, 0.805, 0.824 in the training set (**Figure 2B**), 0.871, 0.829, 0.838 in the validation set (**Figure 2C**), and 0.869, 0.935, 0.835 in the external cohort (**Figure 2D**). Calibration plots were also mapped to assess the accuracy of the survival predictions. The plots show a good agreement between predicted and observed survival probabilities for 1-year, 3-year, and 5-year outcomes, suggesting the nomogram is well-calibrated in the training (**Figure 2E**), validation (**Figure 2F**) and external cohort (**Figure 2G**). The **Figure 3** presents the evaluation of a nomogram for predicting outcomes, including a DCA and a ROC curve. The nomogram in the training set (green line), validation set (red line), and external cohort (yellow line) show higher net benefits across a range of risk thresholds compared to treating all (blue line) or none (purple line) of the patients. This indicates that using the nomogram and validation model provides clinical benefit in decision-making, particularly at lower to moderate risk thresholds (**Figure 3A**). The ROC curve demonstrates the discriminatory power of the nomogram and various individual clinical predictors. The nomogram has the highest AUC (0.835), indicating strong predictive ability. Other individual predictors like AJCC_T (AUC=0.674), AJCC_N (AUC=0.675), and CEA (AUC=0.627) show moderate discrimination, while factors like sex (AUC=0.508) and chemotherapy (AUC=0.525) contribute less to the model’s overall predictive power (**Figure 3B**). Overall, the nomogram outperforms individual clinical predictors, offering robust predictive accuracy and clinical utility. To further evaluate the nomogram model, C-index was also calculated in both training and validation cohorts, and comparisons among the published nomogram models (5, 8, 23, 24) were performed and visualized using forestplot. As shown in **Figure 4**, the nomogram model in the present study outperformed other published models with higher estimate of C-index in both training and validation cohorts.

## 4 Discussion

Colon cancer, a prevalent malignancy of the digestive tract, is primarily addressed through radical resection and comprehensive treatment strategies aimed at enhancing patient quality of life and improving long-term survival rates (25). In the United States, the incidence of colon cancer has seen a notable decline, with a 2.5% annual reduction from 2007 to 2016, accompanied by a 2.1% annual decrease in mortality. Over a broader time frame, the 5-year relative survival rate has increased from 50% in the mid-1970s to 65% by 2016. This improvement can be attributed to advances in traditional chemotherapy and the advent of personalized neoadjuvant chemotherapy (26, 27). However, in many Asian countries, research on post-surgical prognostic factors for colon cancer remains limited. Leveraging the SEER database, this study focuses on identifying prognostic factors for colon cancer in the Asian population post-surgery, highlighting variables such as age, gender, marital status, tumor grade, AJCC staging, CEA levels, and chemotherapy as significant predictors of overall survival.

The historical use of radiation therapy in cancer treatment dates back over a century, with early applications in colorectal cancer surgery reported as early as 1937 (28). The introduction of mega-voltage radiation therapy in the 1980s marked a new era, combining preoperative chemotherapy and intraoperative radiotherapy for advanced tumors (29). However, routine use of adjuvant radiation therapy for colorectal cancer remains unsupported by definitive studies (30). An comparative study assessing adjuvant radiotherapy in addition to chemotherapy showed no benefits on both overall and disease-free survival (31). The present study observed no significant impact of radiotherapy on OS in the Asian population, which could be due to the small sample size of patients receiving radiotherapy, as well as the severity of disease requiring radiotherapy.

Chemotherapy, particularly fluorouracil-based regimens, has proven effective in reducing recurrence and extending survival, especially for stage III colorectal cancer patients. Adjuvant chemotherapy with fluorouracil and leucovorin reduces postoperative mortality by 33% (32). Additionally, a regimen combining fluorouracil, leucovorin, and oxaliplatin has significantly improved 3-year disease-free survival for stage II or III patients (33). Neoadjuvant chemotherapy has shown benefits in downstaging tumors and enhancing disease control rates, with preoperative administration of oxaliplatin and fluorouracil demonstrating improved outcomes (34). In consistent with prior studies, chemotherapy was demonstrated to be a protective factor for OS in Asian colon cancer patients.

The study identified several prognostic factors influencing post-surgical survival in Asian patients. Younger patients, those under 69 years, demonstrated higher survival rates. In contrast, males had a lower chance of survival compared to females. Marital status also played a significant role, with unmarried or separated individuals experiencing worse outcomes than their married counterparts. A previous study (35) has reported tumor size as a prognostic factor in elderly colorectal cancer patients, which was not observed in the present study. This could be due to different patient selection, as our study focused on colon cancer in Asian adults. Apart from patient selection, the effects of tumor size could be partly explained by other variables including tumor grade and stages. In the present study, higher tumor grades and stages were correlated with poorer survival, and elevated CEA levels were associated with reduced survival. The prognostic value of CEA in colon cancer has been well established with extensive literature (36–38). A retrospective cohort analysis (39) concluded that elevated preoperative CEA that normalizes after resection is not an indicator of poor prognosis, whereas patients with elevated postoperative CEA are susceptible to recurrence, especially within the first year after surgery. Therefore, routine evaluation of postoperative, rather than preoperative, CEA is warranted. The significance of marital status aligns with a previous study that linked marital status to disease outcomes (40), indicating that divorced or widowed patients had poorer survival rates. The support and care provided by a spouse may enhance treatment adherence and recovery, whereas single, divorced or widowed patients might encounter greater challenges following surgery. Sufficient data are required to confirm the direct impact of social support on prognosis.

Using SEER database data, this study developed a nomogram model to predict postoperative OS in Asian colon cancer patients. The model demonstrated moderate predictive accuracy and holds potential for guiding clinical decision-making. By examining the incorporated prognostic factors, individualized survival chance can be calculated. Enhanced therapeutic strategies may be given to patients with low predicted survival chances. To our knowledge, the present study reports the first nomogram model for Asian individuals with colon cancer. Furthermore, the nomogram model in the present study outperformed other published models (5, 8, 23, 24) with higher estimate of C-index in both training and validation cohorts. However, limitations include the absence of key clinical data, such as genetic information and lifestyle factors, and the need for external validation. Updates on the details of the latest treatment methods and strategies are also warranted due to the rapid development of cancer treatment. Future research should focus on expanding patient samples through multi-center collaborations, conducting external validations across Asian populations in other areas, and integrating genomic and biomarker data to optimize the model’s predictive accuracy and clinical applicability.

## 5 Conclusion

In conclusion, the current study identified key prognostic factors impacting postoperative overall survival in Asian patients with colon cancer and established a robust prognostic nomogram model. By incorporating variables such as age, gender, marital status, histological type, grade classification, AJCC_T, AJCC_N, and AJCC_M stages, CEA levels, and chemotherapy, the nomogram demonstrated strong predictive capabilities with a C-index of 0.77. The model showed high accuracy in both the training and validation sets, with AUC values consistently indicating reliable predictions for 1-year, 3-year, and 5-year survival. The validation results underscore the nomogram’s potential as a practical tool in clinical settings, empowering healthcare providers with a personalized approach to assess survival outcomes and guide treatment decisions for Asian colon cancer patients. This study contributes valuable insights into the prognostic assessment of colon cancer, potentially improving patient management and outcomes.

## Data Availability

The datasets presented in this study can be found in online repositories. The names of the repository/repositories and accession number(s) can be found in the article/supplementary material. The dataset used for external validation can be requested from the corresponding authors.
